# Automotive Radar Range Spectrum-Based Road Surface Classification by Using Machine Learning

**DOI:** 10.3390/s25226911

**Published:** 2025-11-12

**Authors:** Hima Dominic, Marius Patzer, Marlene Harter

**Affiliations:** Institute for Unmanned Aerial Systems, Offenburg University, Badstrasse 24, 77652 Offenburg, Germany; marius.patzer@hs-offenburg.de (M.P.); marlene.harter@hs-offenburg.de (M.H.)

**Keywords:** automotive radars, surface roughness, AI models, classification, ensemble learning algorithms, Random Forest Classifier

## Abstract

The awareness of different road surface types is crucial for the safe operation of automated vehicles in on- and off-road modes. This paper focuses on the classification of different road surface types using automotive radar and Machine Learning (ML) Artificial Intelligence (AI) models. This analysis is based on the range spectrum of the backscattered radar signals from different road surfaces. The dataset for training and testing is formed by combining the range data from various road surface types. A Random Forest (RF) classifier is built for the identification and classification of four different road surface types. The proposed method is compared using range data obtained from two different mounting positions of the radar. Under dry conditions, a generalization error of 84.5% in the forward-looking position of the radar is achieved. The proposed classifier is also able to distinguish between wet and dry asphalt surfaces with a generalization error of 88.7%.

## 1. Introduction

According to statistics, the number of deaths that occur on the roads in Europe is concerning [1]. To ensure a safe and smooth ride on roads with varying surface types and conditions, the driver must perform appropriate braking maneuvers. Therefore, awareness of road surfaces is a safety-critical Operational Design Domain (ODD) attribute in autonomous driving. Various sensor technologies such as cameras, ultrasonic sensors, and LiDAR are used in automobiles to sense their surroundings for parking assistance [2]. However, a reliable and accurate road surface identification system for safety is still an unresolved task.

As viable solutions to the road surface classification problem, combinations of sensors and AI techniques are being explored in research. A review on road condition monitoring based on AI methodologies and different sensors can be found in [3]. There have been several attempts to use AI for road surface classification, especially using the images of the road surfaces captured by the camera sensors and augmented datasets [4]. In [5], LiDAR technology in combination with supervised learning algorithms is evaluated for road surface classification. Even though image-based classification shows good accuracy, sensor technologies such as cameras and LiDAR are limited in performance by their vulnerability to harsh weather, poor lighting conditions, and any physical obstruction or layer in front of it. Therefore, these technologies are not well suited for road surface classification in real environments. Alternatively, radar sensors show superiority in terms of their range and ability to detect a target irrespective of the weather and lighting conditions.

At present, there are no radar sensors available on the market solely for the purpose of road surface classification. A possible design concept using polarimetry is presented in [6]. However, a polarimetric sensor specifically for this application would be a complex and expensive choice as it requires additional channels. A system that combines a 24 GHz polarimetric radar and a 40 kHz sonar for road surface detection based on neural networks is presented in [7]. This system achieved an average accuracy of 95% by leveraging the sonar’s ability to classify surfaces and the benefits of radar sensors. Despite of the impressive performance, this is an expensive solution with the addition of polarimetry. Further, the 24 GHz radar has less interaction with the physical surface texture compared to a 77 or 79 GHz radar due to its comparatively longer signal wavelength. Similarly, studies have investigated standard automotive radar sensors incorporated with road surface classification capabilities using AI methods. ML-based classification of different material types using 60 GHz radar sensors is explored in [8,9]. Even though they are intended for applications like Human–Computer Interaction (HCI), household robots, etc., they also confirm the possibility of using radar for classifying road surfaces. Classification of surfaces using a 60 GHz pulsed radar mounted on a robot lawn mower is provided in [10]. The classification performances are compared with different models and a high accuracy of above 95% is presented. The high quality of the data acquired from a surface at a very close proximity can be attributed to the high accuracy of these models. However, real-time road surface classification using automotive radar sensors is more challenging than the material classification scenarios considered in these works in terms of data collection and quality of data available for classification. Attempts to use actual radar measurements in combination with AI specifically for road surface classification can be found in [11,12,13], which will be discussed in the following section. These articles differ from each other regarding the used radar sensors, their mounting positions, measured surface types, and the data used for classification and AI models.

This study aims to explore the usability of a commercial 77 GHz Frequency-Modulated-Continuous-Wave (FMCW) automotive radar with minimal modification to its standard mounting position for classifying road surfaces. By leveraging the simplicity and accuracy of traditional ML classification algorithms, solutions are investigated within this work. Considering the random nature of the data which is recorded in real environments, Random Forest (RF) is identified as a suitable choice. RF classifiers [14] are known to provide robustness against data outliers, better generalization capability, and lower overfitting risk. Compared to similar studies, radar range data is used for training and testing the ML model in this paper. Preparation of this range data requires only a few processing steps. Therefore, combining the data generation with the RF classifier can simplify the deployment of the solution by minimizing the computational and power requirements at the edge. The proposed solution adds an additional functionality to a standard automotive radar. This provides the advantage of cost effectiveness as no extra radar will be required for road surface classification.

This paper is organized as follows: Section 2 discusses the related works and emphasizes how they differ from this current study. Section 3 describes the methodology, explaining the data collection, measurement setup, dataset creation, modeling, and classification. In Section 4, a discussion of the classification results is provided. Finally, the conclusion and future perspectives of this work are discussed in Section 5.

## 2. Related Works

In the following section, relevant related works are described. The classification of different surface types using a 60 GHz pulsed radar mounted on a personal mobility device is presented in [11]. They utilized a deep neural network (DNN) with fewer features, which demonstrated high accuracy and optimum inference time. However, the data is collected with the radar looking directly down to the surface from a comparatively close proximity (10 cm), which is not the case with a standard automotive radar. Some of the road surface types considered in this work are less relevant to automobile users. Road surface classification by combining radar and AI is presented in [12], which analyzes the raw backscattered signals from a 77 GHz FMCW automotive radar. Features are extracted from the backscattered received signal using the Mel Frequency Cepstral Coefficient (MFCC) technique to train the classifier. Even though the classifier shows an accuracy of above 97%, this comes at the cost of complex pre-processing steps on the raw radar data. When the amount of data increases, this will increase the computing cost significantly. According to another study conducted in [13], the utilization of a 79 GHz mechanically scanned fan beam radar combined with image processing using a Convolutional Neural Network (CNN) resulted in a high level of accuracy for surface recognition. Two-dimensional images (range cross-range plots) of real road surfaces such as rocky, gravelly, muddy, and uneven countryside roads, as well as a river, were used for the classification. The highly directional fan beam antenna can result in good quality images and better classification. Nevertheless, a mechanically scanning radar will not be suitable for high volume markets like the automotive industry. This solution is expensive in terms of technology and processing. A similar approach with 2D images from radar can be found in [15] where image segmentation techniques are employed. However, this work also employs a scanning beam antenna operating at 79 GHz and the processing steps are computationally intensive.

Unlike the above-mentioned works, this paper uses measurements taken with a 77 GHz automotive radar sensor on real road surfaces for classification purposes. The range data obtained from these measurements are used for road surface classification, which is usually experimented for object classification purposes [16]. This radar is also not specifically optimized for road surface detection. Therefore, the reflections from distributed targets like road surfaces may not serve as very high-quality data for classification. However, the goal is to evaluate this radar’s ability to classify various road surfaces with minimal adjustments to the mounting position and lighter computations. It involves only uncomplicated pre-processing (range data generation) steps and modeling. The fundamentals of using radar for identifying different surface types in most of these investigations are the same, which is explained in the following section.

### Theoretical Fundamentals

The radar-based classification of road surfaces relies on backscattering from different surfaces. The backscattering signal depends on the surface roughness (root-mean-square (rms) height of the surface, correlation length), radar frequency, polarization, angle of incidence, and the effective permittivity of the surface material [13,17]. Surface roughness is one of the important characteristics that differentiates the reflections from different surfaces. An ideally smooth surface reflects an incident plane wave along a single direction according to the law of reflection, which is called specular reflection. As the roughness of the surface increases, more power will be scattered in different directions. For a very rough surface, specular reflections are completely dominated by diffuse reflections.

**Figure 1:**
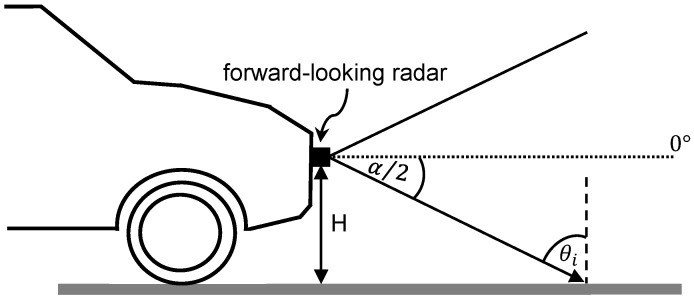
Demonstration of radar signal incidence on a surface.

Based on the Fraunhofer criterion of roughness [18], backscattering from a surface can be distinguished as specular or diffuse [17,18]. According to Fraunhofer criterion, for a ‘rough’ surface, the rms of the surface height distribution (rms height) *s* of the surface satisfies the following inequality:

(1) $$s\geq \frac{\lambda }{32\cdot cos\theta_{i}}$$

where $\theta_{i}$ is the angle of incidence relative to the surface normal and λ is the signal wavelength. A demonstration of a radar mounted on a car at height *H* with an elevation Field-of-View (FoV) α is plotted in Figure 1. The angle of incidence $\theta_{i}$ on the surface is as seen here.

**Figure 2:**
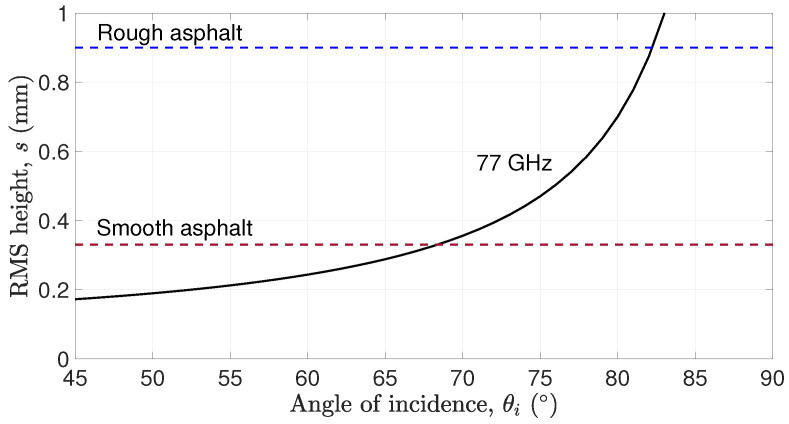
Roughness characterization of asphalt surface by 77 GHz radar as function of incident angle [17].

The calculated *s* for 77 GHz radar frequency is shown in Figure 2, to check the distinguishability of rough asphalt with s=0.9mm and smooth asphalt with s=0.33mm at different angles of incidence. It is clear that both surfaces will appear smooth to the radar when the angle of incidence exceeds 82° (i.e., the *s* values of the corresponding surfaces lie below the 77 GHz radar curve, indicating violation of the Fraunhofer inequality).

Diffuse reflections are significant for road surface classification as they improve the received signal power. According to the above criteria, the ability of a radar to distinguish surfaces improves as its frequency increases.

## 3. Methodology

**Figure 3:**
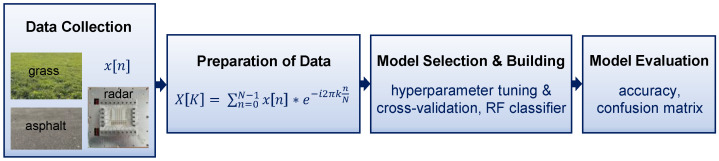
Modeling workflow of proposed methodology.

The goal of this work is to investigate the possibility of using automotive radar for the classification of different road surface types. The workflow to achieve this goal is illustrated in Figure 3. This workflow is adapted to ensure performance generalization and classification accuracy.

### 3.1. Data Collection with Automotive Radar Sensor

For data generation, backscattered signals of radar from different surfaces are acquired. In this investigation, a 77 GHz FMCW radar (Infineon Automotive High resolution radar Evaluation Board CS523, Munich, Germany) with 1 GHz bandwidth is used for the measurements. The elevation Field-of-View (FoV) of this radar is ±30° and the azimuth FoV is ±90°. This radar operates in Multiple Input–Multiple Output (MIMO) configuration with 8 transmit (Tx) and 16 receive (Rx) antennas, i.e., 128 virtual channels. FMCW radars transmit the so called ‘chirp’ during their operation, which is a linear frequency modulated signal. The investigated radar sensor sends out chirps that ramp down from 77 GHz to 76 GHz at a rate of 11 MHz/µs. One measurement frame consists of 8 chirps. These chirps are reflected off the surface and received by the Rx antennas with a shifted frequency. At the receiver, the received signal and transmitted signal are mixed to get the frequency difference as the Intermediate Frequency (IF) signal. This signal is also called the ‘beat’ frequency. It contains information on the range and speed of the target. The beat signal will be digitized by an Analog-to-Digital Converter (ADC) as 512 samples, which is the acquired raw radar data in the time domain. Thus, each measurement provides a radar cube with dimensions of 512 × 8 × 128, i.e., number of samples × number of chirps × number of virtual channels.

**Figure 4:**
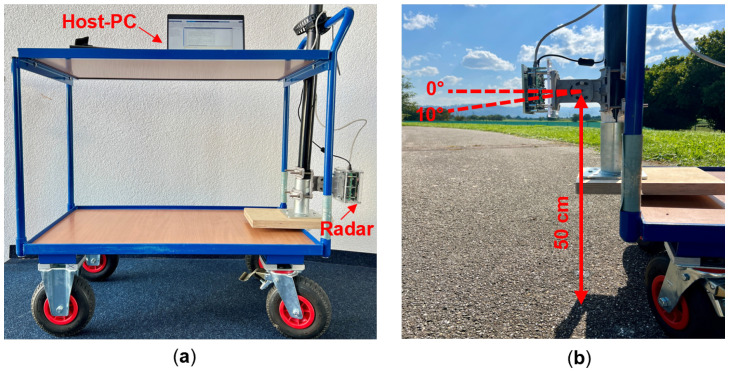
(**a**) Measurement setup for data collection from different road surface types with the radar mounted on a trolley cart and host-PC. (**b**) Mounting positions of the radar with two mounting angle possibilities.

Figure 4a shows the measurement setup with this radar. As can be seen in Figure 4b, the radar is mounted at a height of 50 cm from the ground and it can be rotated in the vertical direction. The main goal of this work is to investigate if a standard automotive radar can be used additionally for road surface classification without the need of a separate sensor. Since the forward-looking position of automotive radars is not optimized for road surface classification, it is necessary to compare its performance with a different mounting angle of the radar sensor. However, the inclination of the radar towards the ground will lead to a reduction in the maximum detection range of the radar. Based on our experiments with different mounting angles, range spectra from different surfaces with 10° inclination of the radar already showed noticeable differences. Therefore, 10° inclination is chosen without reducing much of the maximum range of the radar.

**Figure 5:**
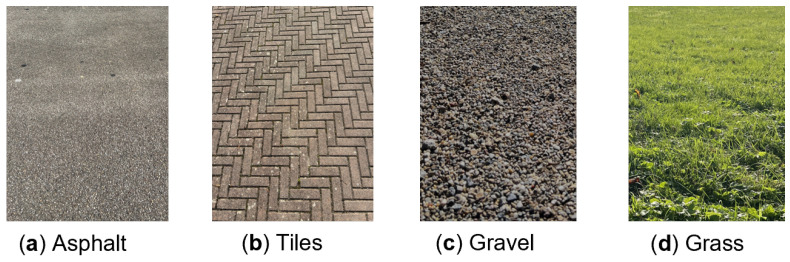
Road surface types considered for classification.

Measurements are performed at forward-looking (0°) and 10° inclined (towards the ground) position of the radar, as indicated in Figure 4b. These two vertical orientations were investigated to identify a best solution for classification. The data was collected from four types of road surfaces such as asphalt, tiles (paver tiles), gravel, and grass under dry conditions, which are demonstrated in Figure 5a–d. These surface types are chosen such that it includes typical types in on-road and off-road conditions. Even though grass is not a typical road surface, it is mainly used for testing and comparing different reflection behaviors. An equal number of measurements (300 measurements with single frame per measurement) is conducted on each surface from three different locations. Within each location, the measurements are carried out at uncorrelated positions to ensure the randomness of the data samples.

### 3.2. Preparation of Data

From the samples of the IF signal collected in the previous step, Fast Fourier Transform (FFT) is calculated. The amplitude spectrum (amplitude vs. frequency) from the FFT is converted to the range spectrum by changing the frequencies into the corresponding range values. To calculate range *d* corresponding to the frequency *f*, the following equation is used:

(2) $$d=\frac{f\cdot T\cdot c}{2\cdot B},$$

where *T* is the duration of the chirp, *c* is the speed of light, and *B* is the bandwidth of the chirp.

The dataset for the classification problem is formed using these range spectra. To have a proof-of-concept, it is necessary to use a dataset with the range spectra corresponding to the reflections only from each surface type. The exclusion of reflections from other cars and road side objects is done by gating the range. According to the elevation Field-of-View of the radar, the range at which the beam hits the ground is estimated. With respect to this, the range spectrum used for training and testing is prepared by limiting the range. As it is difficult to avoid all possible obstacles in real environments, this work also aims to verify if the classification of surfaces is possible even in the presence of such reflections in the range spectra.

As there are 128 virtual channels, it is possible to get 128 range spectra from one measurement. Therefore, the dataset has 300 × 128 range spectra for each class. This ensured a large number of data samples per surface type. Two datasets are prepared from measurements at two different orientations of the radar. Each dataset consists of 153,600 data samples from all classes. The labeling of the data is performed based on the ground truth.

**Figure 6:**
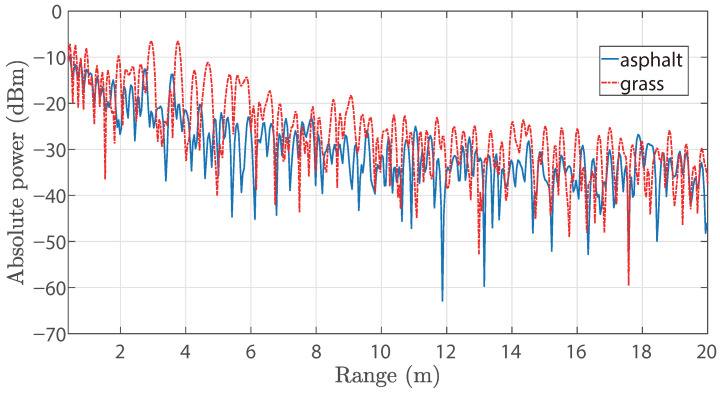
Exemplary range spectrum from asphalt and grass measurements with forward-looking radar (i.e., 0°).

Grass and asphalt surfaces are considered as an example to compare the range spectra, which is shown in Figure 6. It is evident that the range spectrum of grass shows higher reflection power due to comparatively rougher appearance and asymmetric distribution. This also confirms that surfaces can be distinguished by their range spectrum.

### 3.3. Classification with Random Forest Classifier

Traditional ML models are considered for the classification of the range spectrum from different road surfaces. The Support Vector Machine (SVM) classifier was compared with the RF classifier, but the accuracy of the SVM was inadequate. Therefore, the RF classifier was chosen for the classification of the road surfaces using range spectra. This supervised learning algorithm is also an ensemble learning algorithm made of multiple decision trees. The predictions of several decision trees will be combined to improve the robustness of the algorithm. RF classifier’s ability to handle data outliers has favored the classification of range spectra with possible data outliers in the form of high amplitude reflections from unavoidable road objects.

A baseline model is built with default hyper-parameter values of the estimator. The dataset from the forward-looking radar is split into 70% training (i.e., 107,520 data samples) and 30% test (i.e., 46,080 data samples) data, which are class-balanced subsets of the dataset. After evaluating the model, the accuracy is found to be 82%.

In order to get the best model without the risk of overfitting and to evaluate the generalization capability of the model, hyper-parameter optimization and cross-validation (CV) are performed. This is done with a nested CV approach, where a k-fold CV procedure for model hyper-parameter optimization is nested inside the k-fold CV procedure for model selection. Five-fold cross-validation is performed for model selection and a grid search algorithm [19,20] is used for hyper-parameter optimization. Nested CV is performed on both datasets. Hyper-parameters, such as the number of decision trees (n_estimators), maximum depth of the tree (max_depth), and the minimum number of samples required to split an internal node (min_samples_split), are considered for the optimization. The optimized hyper-parameter values of the model for 0° dataset are n_estimators = 500, max_depth = none, and min_samples_split = 10. Note that if the maximum depth value is none, then the nodes are expanded until all leaves contain less than min_samples_split samples. For the 10° inclination dataset, the optimized hyper-parameters are n_estimators = 500, max_depth = none, and min_samples_split = 2.

The models resulting from nested cross-validation are used for prediction on the test data, thereby evaluating the model.

## 4. Results and Discussion

**Figure 7:**
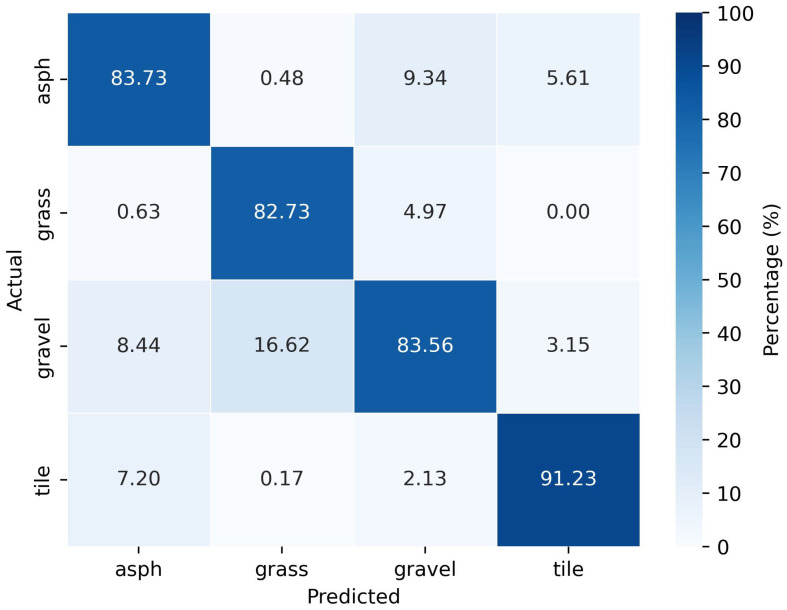
Confusion matrix of classification with dataset from forward-looking radar (0°).

From the nested CV procedure, the generalization error corresponding to the model with the forward-looking radar dataset was found to be improved to 84.5%. The optimized model is used to evaluate the performance. The resulted confusion matrix is plotted in Figure 7, where ‘asph’ implies asphalt.

According to the confusion matrix shown in Figure 7, tiles are predicted with the highest accuracy of 91.23%. This may be due to the uniform pattern of the tiles and the significant reflections they cause compared to the other surface types. A high error rate of 16.62% is observed in the prediction of gravel and it is incorrectly predicted as grass. This can be attributed to their similar surface irregularities. Further, the similar appearance of asphalt, gravel, and tiles might have contributed to some incorrect predictions of these classes as well. Grass and asphalt, as well as grass and tiles, can be distinguished quite well by the implemented model.

**Figure 8:**
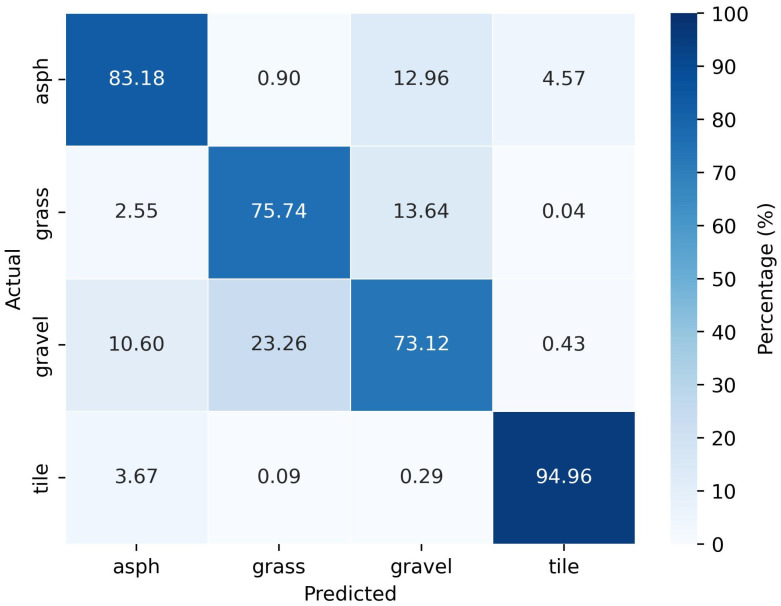
Confusion matrix of classification with dataset from 10° inclination.

For the realized model with the 10° inclination dataset, the generalization error is found to be 82%. The evaluation of the optimized model resulted in the confusion matrix demonstrated in Figure 8. Similar to the results from the model with the 0° dataset, the prediction on tiles is quite impressive with an accuracy of almost 95%. Compared to the model with the 0° dataset, the rate of incorrect predictions between tiles and asphalt classes was reduced to 3.67% and 4.57%. Grass is well separated from tiles and asphalt, as only a few wrong predictions are made, similar to the model with the 0° dataset. However, the incorrect predictions between asphalt and gravel, as well as between grass and gravel, are quite high. This could be due to the similarities in the reflections from these groups of surfaces. Contrary to the model with the 0° dataset, tile and gravel are well distinguishable with the 10° inclination dataset.

In order to have better compare the classification performance with the two datasets, *F*1-score, *recall*, and *precision* values are estimated. The definition of *F*1-score is as follows:

(3) $$F1=\frac{2\times precision\times recall}{precision+recall},$$

with precision and recall calculated as

(4) $$precision=\frac{T_{p}}{T_{p}+F_{p}};\;recall=\frac{T_{p}}{T_{p}+F_{n}};$$

where $T_{p}$, $F_{p}$, and $F_{n}$ are the number of true positives, false positives, and false negatives from the confusion matrix. The estimated values of these performance metrics are presented in Table 1 and Table 2.

Clearly, the optimized models for the 0° and 10° inclination datasets show slightly different abilities in distinguishing different surface type combinations. Yet the optimized model with the 0° dataset exhibits superiority in terms of generalization error. This model has a higher percentage of accuracy and better values of other performance metrics in each category. Therefore, the usage of a model with data from a forward-looking position (0°) can be recommended. This offers an advantage, as no radar sensor inclination is needed for road surface classification.

### Influence of Wet Surface Conditions

**Figure 9:**
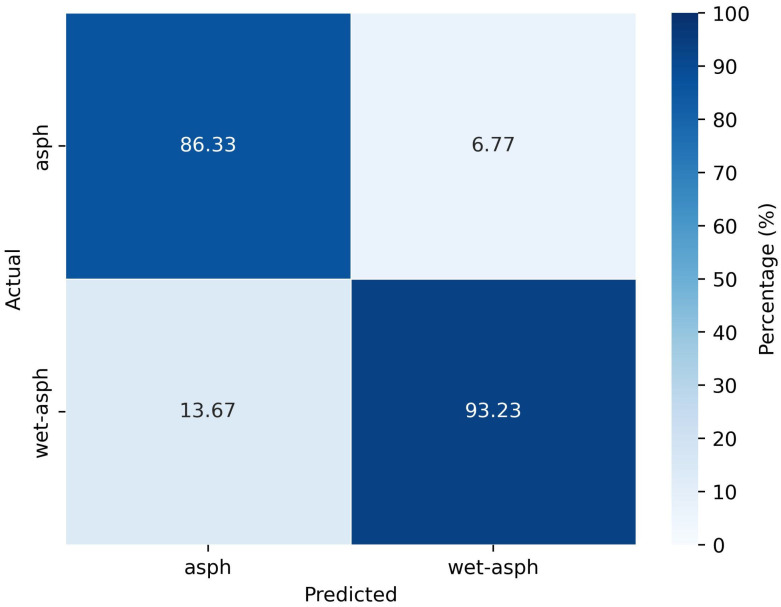
Confusion matrix of classification with dry and wet asphalt data from forward-looking position of radar.

To analyze the classification performance under wet conditions, extended research wass conducted by considering asphalt surface. As the model with data from the forward-looking position of the radar performed better in classifying different surfaces, only this radar position is considered for the analysis. Measurements are conducted on a wet asphalt surface and a dataset with dry and wet asphalt surfaces is prepared according to the steps explained in Section 3.1 and Section 3.2. The dataset is used for classification with the RF classifier and a generalization error of 88.7% is obtained. The evaluation of the model resulted in the confusion matrix as shown in Figure 9. Here, ’asph’ represents the dry asphalt surface.

From the confusion matrix, it is clear that the incorrect predictions between wet and dry asphalt have a smaller percentage and the accuracy of correct predictions is high. The incorrect predictions between wet and dry asphalt could be due to the imperfections in the experimentally created wet asphalt surface, which might contain patches of dry asphalt regions. Therefore, it can be concluded that properly wet asphalt can be distinguished well from dry asphalt surfaces due to the difference in roughness of the surfaces.

Overall, this work demonstrates that the road surfaces can be classified just by using range measurements from standard automotive radars. As mentioned earlier, the dataset was prepared using range spectra within a limited range to capture reflections only from road surfaces. However, it was not possible to have a completely controlled environment with no other objects. The high classification performance ensures the robustness of the proposed model against reflections from such unavoidable objects in the vicinity of the radar.

## 5. Conclusions

An investigation of the possibility of using range spectra for classifying road surfaces with an RF classifier was established. Asphalt and tiles can be easily distinguished from grass in both the forward-looking (0°) and 10° inclined positions of the radar. Tiles are well recognizable from all other surface types according to the results from both positions. Overall, both models presented are capable of distinguishing certain surface classes with high accuracy (above 90%) and others with reasonable accuracy. From the results, it can be concluded that a specially designed radar is not necessary for classifying road surfaces. It was also demonstrated that even the forward-looking position of the radar could be favorable for road surface classification. Promising classification performance is obtained in distinguishing wet and dry asphalt in the forward-looking position of the radar with an accuracy of 88.7%.

As future work, feature selection will be implemented to analyze if an improvement can be achieved. It is also planned to expand the dataset with additional road surface types and analyze the separability between different groups of surfaces. Deep learning models such as CNN using range cross-range 2D images will be analyzed in the next phase.

## Figures and Tables

**Table 1:** Performance metrics for classification with dataset from forward-looking radar (0°).

Predicted Class	Precision	Recall	F1-Score
Asph	0.84	0.86	0.85
Grass	0.83	0.95	0.89
Gravel	0.84	0.69	0.75
Tile	0.91	0.91	0.91

**Table 2:** Performance metrics for classification with dataset from 10° inclination.

Predicted Class	Precision	Recall	F1-Score
Asph	0.83	0.83	0.83
Grass	0.76	0.86	0.80
Gravel	0.73	0.62	0.67
Tile	0.95	0.96	0.95

## Data Availability

The datasets presented in this article are not readily available because the data are part of an ongoing study and due to privacy. Requests to access the datasets should be directed to the corresponding author.

## Abbreviations

The following abbreviations are used in this manuscript:

AI	Artificial intelligence
ADC	Analog-to-digital converter
CNN	Convolutional neural network
CV	Cross-validation
DNN	Deep neural network
FFT	Fast fourier transform
FMCW	Frequency-modulated continuous wave
FoV	Field-of-view
HCI	Human–computer interaction
IF	Intermediate frequency
MFCC	Mel frequency cepstral coefficient
MIMO	Multiple input–multiple output
ML	Machine learning
ODD	Operational design domain
RCS	Radar-cross-section
rms	root-mean-square
RF	Random forest
Rx	Receive
SVM	Support vector machine
Tx	Transmit
